# Effects of structured patient education on knowledge level and INR control of patients receiving warfarin: Randomized Controlled Trial

**DOI:** 10.12669/pjms.342.14216

**Published:** 2018

**Authors:** Ebru Baysal, Tulay Sagkal Midilli

**Affiliations:** 1 Ebru Baysal, Research Assistant, Department of Fundamentals Nursing, Ege University School of Nursing, Izmir, Turkey; 2 Tulay Sagkal Midilli, Assistant Professor, Department of Fundamentals Nursing, Manisa Celal Bayar University School of Health, Manisa, Turkey

**Keywords:** International normalized ratio, Nursing, Patient education, Randomized controlled trial, Warfarin

## Abstract

**Objective:**

To determine the effects of patient education about the safety of warfarin therapy on related-knowledge levels and on International Normalized Ratio (INR) control.

**Methods:**

In the study, randomized controlled experimental study design was used. It was conducted between September 2014–March 2015 with 63 patients who use warfarin at least two months at cardiology and cardiovascular surgery outpatient departments of two different hospitals in Manisa. Participants in the intervention group received one-to-one education about the safety of warfarin therapy and a booklet. Participants in the control group received usual care. Patients’ warfarin knowledge levels in both groups were measured three times at monthly intervals.

**Results:**

Before education warfarin knowledge levels were inadequate in intervention group, but it was higher after education and reached a good level. No significant difference was found between the International Normalized Ratio controls of the two groups. No significant relationship was found between pre- and post-education warfarin knowledge levels and the INR number in the therapeutic range.

**Conclusion:**

One-to-one education supported by written and visual material was effective in increasing patients’ warfarin knowledge levels.

## INTRODUCTION

Today, morbidity and mortality rates from thromboembolic diseases are high. Anticoagulants are used in the treatment and prophylaxis of these diseases. Warfarin is the most widely prescribed oral anticoagulant in the World.^1^,^2^ However, because of the narrow therapeutic index and life-threatening complications, patients must be regularly and continuously followed up.^3^ The warfarin dose is set according to the International Normalized Ratio (INR) level in the blood. The target range of INR is 2-3 in patients using warfarin and can vary with indications.^4^,^5^ In the first stages of treatment, INR is monitored at frequent intervals, but after the INR reaches the desired level, monitoring is carried out routinely once every four weeks.^4^ Studies have shown that when the INR level exceeds the therapeutic range the risk of bleeding increases, and when it falls below 2, the risk of thromboembolism increases.^1^,^4^,^5^

Although warfarin is widely used, it can have life-threatening adverse effects when knowledge of patients about medication interaction with food and with other drugs, and laboratory test monitoring are inadequate.^6^ The most important and most often seen adverse effect of warfarin treatment is bleeding.^1^

A patient’s level of knowledge concerning the drug plays a key role in the effective and safe use of warfarin. In the Joint Commission International (JCI) National Patient Safety Goal (NPSG) guideline for 2014 education on treatment for patients taking oral anticoagulants was recognized as a vital factor.^7^ Previous studies have reported that a positive relationship between patients’ anticoagulant knowledge levels and the INR number in the therapeutic range^8^-^11^, and adherence to treatment.^10^,^12^ For this reason there is a need for effective education programs to increase patients’ knowledge concerning warfarin and to keep it up to date. Education and information services provided to patients using anticoagulants will reduce the costs of treatment by reducing risks such as thromboembolism and bleeding.^10^ Educating the patients who use warfarin is one of the important responsibilities of the nurses’ counseling and educational roles as much as doctors’ roles. In a study by Johnson et al. (2010), it was determined that patient education improves patients’ attitudes and medication adherence, which emphasized the importance of nursing counseling and education programs.^13^ When planning education, individual characteristics and needs must be kept in mind, and group education in addition to individual sessions and the use of written and visual material will increase the effectiveness of the education.^14^ In order to make the information permanent, oral and written materials were used (PPT Presentation and Booklet) in the education. To assess the persistence of the information, the patient’s level of knowledge measurements were repeated immediately after the education, one month and two months later and the patient’s questions after the education were answered on the phone.

The primary aim of this study was to determine the knowledge of patients using warfarin concerning its use, and the effects of education on the safety of warfarin therapy on patients’ knowledge levels and INR control. A secondary aim was to contribute to development an education program and booklet for patients using warfarin in health institutions.

### Hypotheses

1. Are education programs effective in improving patients` warfarin knowledge level and INR control?
2. Is there an association between patients` warfarin knowledge and INR control?

## METHODS

**Fig.1:**
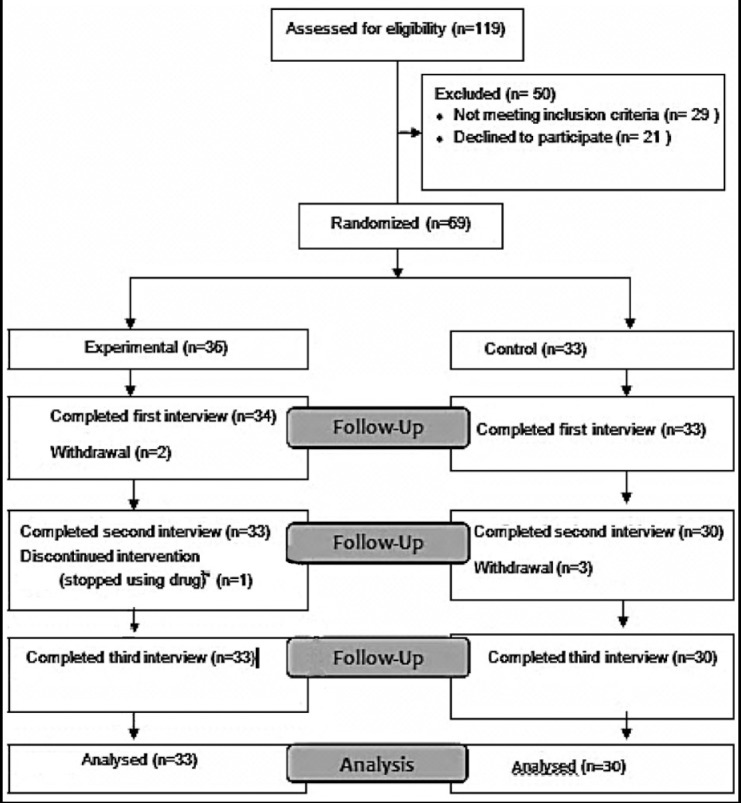
CONSORT 2010 Flow Diagram.

The study had a pre-test post-test randomized controlled trial design. The sample included 63 patients using warfarin between September 2014 and March 2015. It was carried out at the cardiology and cardiovascular surgery outpatient clinics of two different hospitals in Manisa. A total of 119 subjects were screened for eligibility of which 29 failed to meet the criteria and 21 patients refused to participate in the study. Sixty nine patients who met the eligibility criteria accepted to participate in the study and were randomized. A total of 69 patients were initially included in the study, 36 in the experimental group and 33 in the control (Fig.1).

Patients were included into the study that had been using warfarin for at least two months, who were between 18 and 65 years old, who were literate, and who accepted voluntarily to take part in the study. Patients were excluded from the study that had previously had a structured education about warfarin, which had a seeing or hearing disability, who had a cognitive or sensory disorder, or who had a disability affecting oral communication.

### Sample size and statistical power considerations

The sample size was calculated using the SPSS program, based on Khan et al.^9^ It was calculated as 25 patients for the intervention group and 25 for the control group with a post-education increase in warfarin knowledge levels of 25%, Type-I error rate of 0.05 and 80% power. A total of 33 patients were included in the intervention group and 30 in the control group.

### Randomization

The participants were randomly allocated to either the intervention group (n=33) or the control group (n=30) according to their hospital protocol number. Lots were drawn by the clinic nurse who was independent from the study, and those with odd numbers of protocol numbers were placed in the control group and those with even of protocol numbers in the intervention group. Before the randomization, the researcher explained the study’s purpose and procedures to patients in the control and intervention groups.

### Instrument

### Data collection

Patients using warfarin due to various diagnoses refer to the hospital for INR measurement at least once a month. The patients were able to contact the cardiology and cardiovascular surgery outpatient clinics between the scheduled follow-up visits. The researcher met with the all patients who were admitted to outpatient clinic two day a week. After the eligibility criteria of the patients were reviewed, initial assessments were completed and the participants were subsequently randomized. An appropriate room in clinic was provided to the researcher for meeting with patients by hospital management.

Data were collected via two questionnaires between September 2014 and March 2015 at four time points for experimental group and at three time points for control groups. These were a Patient Identification Form and a Warfarin Knowledge Assessment Form, and were prepared by the researcher in based to with the literature.^2^,^3^,^6^,^8^,^10^,^11^

***The Patient Identification Form*** consisted of 20 questions on information on sociodemographic characteristics and illness-related information such as age, gender, chronic illness and medications used, consumption of alcohol and tobacco, warfarin dose, duration of warfarin use, and the existence of warfarin adverse effects.^2^,^3^,^6^,^8^,^10^

***The Warfarin Knowledge Assessment Form*** consisted of 30 questions. These questions covered basic information for drug treatment such as the drug dose, the duration of drug use, adverse effects of the drug, drug-drug and drug-food interaction, drug use in pregnancy, dental care and treatment, laboratory monitoring, exercise and diet.^2^,^3^,^6^,^8^,^10^,^11^ Each correct answer scored 1, and each wrong answer or a “don’t know” response scored 0. The last two questions in the last section on drug use related to pregnancy and nursing a child, and so these were answered only by women. Patients’ raw information scores were between 0 and 30. Those scoring 0-15 were accepted as having a low level of information, those scoring 16-21 a moderate level, and those scoring 22-30 a high level of information. Cronbach’s alpha of the form was 0.86. The references should be added. 

### Intervention

### Booklet

A booklet was developed with the aim of educating patients on the safe use of warfarin. This booklet contained basic information on the use of warfarin such as how the drug should be used, its adverse effects, drug-drug and drug-food interactions, dental care, exercise, and laboratory monitoring. Expert opinion was obtained from ten persons (two cardiology doctors, two cardiovascular surgery doctors and six nursing instructor) before the study for the booklet and the questionnaire forms. In order to evaluate the comprehensibility of the questionnaire forms and the booklet, a pilot study was conducted with ten patients. Based on their feedback, some words and sentences were changed for clarity.

### Education program

At the first interview, the questionnaires were applied to both groups. Afterwards, patients in the intervention group were given a power point presentation about the safety of warfarin therapy of approximately 45 minutes, and a one-to-one education session. After the education session, the warfarin knowledge assessment form was applied to patients once more and the education booklet provided was prepared based on the literature.^15^,^16^ Patients were asked to read the education booklet at home. A phone number was given to patients for their questions about the booklet and researcher answered them during the study.

No intervention was performed on the patients of the control group. The usual care for patients with warfarin usage in Turkey is informing by a doctor but structured education not available in all hospitals. The warfarin knowledge evaluation form was applied again to the patients of each group in the first and second months, and the INR values for those dates were obtained from hospital records. Any harm was not observed in the control group patients due to study. One to- one education and education booklet were given to patients in the control group not to violate the right to information of them after the study ended.

### Data analysis

Data were analyzed using the Statistical Package for Social Sciences (SPSS 21) software for Windows. Descriptive statistics were used to describe the socio-demographic and disease characteristics of the sample (mean, SD). In order to evaluate patients’ INR measurements, numerical and percentage distributions and chi-square analysis (χ^2^) was used. In the evaluation of mean warfarin knowledge scores, the Independent Samples t-test was used. In order to evaluate the correlation between mean knowledge scores and INR control, correlation analysis was used.

### Ethical considerations

The research was approved by the faculty’s ethics committee (Approval No. 20478486-309). The study conformed to the principles outlined in the Helsinki Declaration. Informed consent was obtained orally and in writing from the patients taking part in the study. The information included the purpose and procedures of the study, the voluntary nature of their participation and the option to withdraw at any time.

## RESULTS

### Study sample

Baseline characteristics of patients in each of the study groups were examined. It was found that the groups were similar except for the type of chronic diseases in the intervention group. The mean age of the patients was 49.9±11.8 years, most (70%) were educated to primary level, and half (51%) were using warfare because of heart valve replacement. Half of the patients (52%) had comorbidity, the most frequent of which (25%) was hypertension. Nearly all the patients stated that they used warfarin in a regular way.

### The effects of the education program on warfarin knowledge level and INR control

The distribution of patients’ INR values is shown in Table-I. Patients in the intervention and control groups were similar to INR values of all measurements (p>0.05). Except to the first and third measurements, the INR values of more than half of the patients in the intervention groups were within the therapeutic range. In the control group, the INR values of more than half of the patients were within the therapeutic range except to the first and fourth measurements is shown the mean warfarin knowledge scores distribution of patients before and after education is shown in Table-II. Before education, the mean knowledge scores of the intervention group were 14.2±4.6, and the mean score of the control group was 14.1±6.3. Before education, there was no significant difference between the two groups with regard to patients’ mean knowledge scores (p>0.05). In the final interview, two months after the education, the mean knowledge score of patients in the intervention group was 22.5±3.7, and the mean knowledge scores of patients in the control group had risen significantly, and was 16.2±6.4. No statistically significant correlation was found between the mean knowledge scores of the patients in the intervention and control groups measured before and after education and the INR number in the therapeutic range (p>0.05) (Table-III).

**Table-I:** Distribution of INR levels by time of INR measurement of patients in the interventional and control groups (n=63).

Measurements	INR Levels	Interventional (n=33)	Control (n=30)	Total n=63	p value*

		n (%)	n (%)	n (%)	
	<2	11 (33)	11 (37)	22 (35)	
1st measurement	2-3	15 (46)	11 (37)	26 (41)	0.763
	>3	7 (21)	8 (26)	15 (24)	
	<2	7 (21)	10 (33)	17 (27)	
2nd measurement	2-3	17 (52)	16 (53)	33 (52)	0.310
	>3	9 (27)	4 (14)	13 (21)	
	<2	10 (30)	11 (37)	21 (33)	
3rd measurement	2-3	13 (39)	15 (50)	28 (44)	0.269
	>3	10 (31)	4 (13)	14 (23)	
	<2	11 (33)	13 (43)	24 (38)	
4th measurement	2-3	19 (58)	12 (40)	31 (49)	0.348
	>3	3 (9)	5 (17)	8 (13)	
	<2	9 (27)	15 (17)	14 (22)	
5th measurement	2-3	18 (55)	16 (53)	34 (54)	0.423
	>3	6 (18)	9 (30)	15 (24)	
	<2	7 (21)	5 (17)	12 (19)	
6th measurement	2-3	18 (55)	22 (73)	40 (64)	0.238
	>3	8 (24)	3 (10)	11 (17)	

*Abbreviations:* <2: Subtherapeutic, 2-3: therapeutic, >3: Supratherapeutic,

* Chi-square analysis.

**Table-II:** Comparison of mean warfarin knowledge scores of interventional and control groups (n=63).

Measurement Time	Interventional (n=33)	Control (n=30)	Test

	mean (SD)	mean (SD)	
Before instruction	14.2± 4.6	14.1± 6.3	t= 0.062^‡^ p= 0.951
After instruction*	21.37 ± 3.89		
1 month after instruction	21.9 ± 3.5	14.9 ± 6.0	t= 5.673^‡^p=0.001**
2 months after instruction	22.5 ± 3.7	16.2 ± 6.4	t= 4.801^‡^p=0.001**

* Measurement made immediately after education,

** p<0.05,

‡ Independent Samples t-test.

**Table-III:** Correlation between patients’ mean knowledge scores and INR control (n=63).

Warfarin knowledge score	Number of INR in Therapeutic Range	

	Interventional (n=33)	Control (n=30)
Average score before education	r: 0.197 p: 0.271	r: 0.124* p: 0.514
Average score after education	r: 0.112 p: 0.533	r: 0.217 p: 0.250

* Spearman correlation.

## DISCUSSION

Achieving success with warfarin treatment necessitates effective INR control in order to prevent complications such as thromboembolism and bleeding.^17^ In this study it was found that the number of patients with a therapeutic INR values before and after education was low in both groups. An increase in INR control was expected along with the increase in knowledge levels of patients in the intervention group after education, but the results showed no significant difference between the intervention and control groups. In studies to determine INR control in patients using warfarin, the percentages of patients with therapeutic INR values were variable. In a study by Kalra et al. (2000), 61% of patients were found to have INR values within the therapeutic range.^18^ This percentage was found to be 61% in Nelson et al. (2015) and 67% in Oramasionwu et al. (2014).^19^,^20^ The present study confirms findings of earlier studies done in Turkey that INR control is better in patients in studies in other countries.

Patient knowledge level plays an important part in the prevention of the serious complications which can be seen with treatment. The incorrect use of medication by patients whose knowledge levels are inadequate increases the risk of thromboembolism and bleeding.^21^ Mean scores of patients in the intervention and control groups of the study were found to be similar before education, while in measurements made one and two months after education it was found that the mean knowledge scores of patients in the intervention group were significantly higher. Results of studies to determine the knowledge levels of patients using warfarin show that knowledge levels are low.^8^,^11^,^22^ Two studies also report that patients’ warfarin knowledge levels were at a moderate level.^12^,^23^

Because no correlation was found between patients’ warfarin knowledge levels and the INR number within the therapeutic range, it was concluded in the study that INR control was not affected by warfarin knowledge levels (p>0.05). A longer follow-up period may be necessary to determine behavior changes resulting from knowledge acquired by patients following education and their reflection in INR measurement results. In addition, in situations which are out of the control of the patient or which have a genetic cause such as warfarin resistance, INR values may be affected, and even if patient adherence to treatment is adequate, INR control may be insufficient.^12^ In some studies, positive correlations have been found between patients’ knowledge levels and anticoagulant control and drug adherence and INR numbers within the therapeutic range, and it has been seen that the INR control of patients whose knowledge was at an adequate level was better than that of that of patients whose knowledge level was inadequate.^8^,^9^,^10^,^11^ However, other studies are in agreement with the present study in showing that patients’ INR control is not affected by knowledge level.^24^,^25^

### Limitations of the study

Patients over the age of 65 were excluded from the study because their literacy rates were low, in order to reduce the effect of education. Also, a standard questionnaire was not used to measure patients’ warfarin knowledge levels. For these reasons, the results should be relevant to the population in this study. The fact that the one-to-one education and the application of the questionnaire took approximately 60 minutes and patients’ unwillingness to participate in the application of the questionnaire in the first and second months resulted in the loss of some patients.

## CONCLUSIONS

After the education, patients in the intervention group on the safety of warfarin therapy, patients’ warfarin knowledge levels increased by approximately 50%, and it was found that the warfarin knowledge levels of the intervention group were significantly higher than those of the control group. There was no significant difference between INR control in patients in the intervention and control groups. An increase in INR control was expected as a result of an increase in patients’ knowledge of warfarin, but no correlation was found between INR control and warfarin knowledge levels. One to one patient education and the individualized education should be given by determining patients’ individual differences and lack of information. Health institutions should implement regular patient education programs and booklets about the safety of warfarin to prevent life-threatening complications. Achieving success with warfarin treatment is required effective INR control in order to prevent the complications.^17^ A longer follow-up period may be necessary to determine behavior changes resulting from knowledge acquired by patients following education and their reflection in INR measurement results.

## Clinical Trial Protocol Registration and Results System ID

CBU-2015-02.

## Trial registration number

NCT02757482 (Clinical Gov Trial).
